# In the perception of the Olympic movement and gender equity in sport, are gender and sport practice determining factors?

**DOI:** 10.3389/fspor.2025.1564617

**Published:** 2025-03-31

**Authors:** Hussein Muñoz-Helú, Luis Felipe Reynoso-Sánchez, Karla Noelia Cruz-Morales, Ciria Margarita Salazar-C, Leonardo Jose Mataruna-Dos-Santos

**Affiliations:** ^1^ Department of Economic-Administrative Sciences, Autonomous University of Occident, Los Mochis, Mexico; ^2^ Research Centre for Physical Culture Science and Health, Autonomous University of Occident, Culiacan, Mexico; ^3^ Department of Social Sciences and Humanities, Autonomous University of Occident, Los Mochis, Mexico; ^4^ Faculty of Education Sciences, University of Colima, Colima, Mexico; ^5^ School of Management, Canadian University Dubai, Dubai, United Arab Emirates

**Keywords:** Olympism, Olympic values, women athletes, exercise sciences, university students, gender equity

## Abstract

**Background:**

The Olympic Movement (OM) has evolved considerably, with the modern Olympic Games as its main emblem in the world. Within its most recent agenda, issues such as sustainability, fair play, however, inclusion and gender equity have gained greater relevance, especially through Olympic Agenda 2020, its subsequent recommendations in Agenda 2020 + 5 and the strong influence of the Sustainable Development Goals (SDGs) promoted by the United Nations (UN). This study analyzes the conditional role of gender and sports practice level in moderating the influence of the Olympic Movement's perception on attitudes toward women's participation in sports among Mexican university physical education, sports, and exercise sciences students.

**Method:**

A cross-sectional, correlational-descriptive design was employed with a sample of 415 students (33.5% women) from 15 higher Mexican education institutions. Data collection utilized the Olympism Vision and Its Educational Repercussions Questionnaire and the Scale of Attitudes Toward Women's Participation in Sports. A double moderation analysis examined how gender and sports practice level moderated the relationship between Olympic Movement perceptions (threats, values, significance) and attitudes toward women's sports participation.

**Results:**

Women exhibit greater knowledge of Olympism than men, as do high-level sports practitioners compared to their less active counterparts. Women also express a stronger positive perception of women in sports but report lower perceptions of equity and social support for women in sports compared to men. Double moderation analyses revealed that gender and sports practice level significantly shaped the relationship between perceptions of the Olympic Movement and attitudes toward women's sports participation. Women at low-to-moderate sports participation levels perceived greater threats to gender equity and more strongly identified with the Olympic Movement's values in promoting women's participation. Elite athletes of both genders recognized gender inequities within sports systems, while non-active participants linked women's participation to Olympic ideals over systemic actions.

**Conclusion:**

The results underscore the need for targeted policies and educational strategies to enhance gender equity and support women's sports participation. Promoting Olympic education within academic and extracurricular frameworks could strengthen critical awareness of human rights and sports values, countering stereotyped narratives and fostering equitable opportunities in sports.

## 1 Introduction

The Olympic Movement (OM) and its philosophy has been characterized by its constant evolution with the modern Olympic Games (OG) being the most recognized emblem of Olympism worldwide. Over the past decades, fair play, sustainability, inclusion and gender equity have been extremely important themes in Olympism, driven most strongly by Olympic Agenda 2020 (1) and reinforced in the recommendations issued by Olympic Agenda 2020 + 5 (2).

Considerations of respect for human rights were an important part of the Olympic Movement's development since the beginning of the Modern Olympic Games. For the International Olympic Committee (IOC), the promotion of peace, solidarity and brotherhood among nations is of great relevance (3). Along with these premises, the struggle for gender equality is a constant activity in the promotion of the Olympism values. Avoiding discrimination in each of the sport fields must be a shared responsibility by each of the actors from the different competition areas (4) and eliminate all gender violence in the sports and competitive system, in a study carried out by (5), women athletes in aesthetic sports face specific challenges in terms of confidence and concern for their integrity and safety than those in other sports.

Womens' inclusion in the Olympic Games has increased considerably since its first competition in 1900 with the participation of 22 athletes. By 2016, the IOC issued 25 recommendations to encourage equal participation in Olympic and Federated sports. After the Olympic Games held in Rio 2016 and Tokyo 2020–21 (held a year later than agreed as a result of the COVID-19 pandemic), the IOC in Paris 2024 reached the 50–50 forecast in sports participation, in the rest of the tasks, especially those of leadership, the Olympism governing board considers fulfilling in the Olympic Games of Los Angeles 2028 (6).

It is important to observe not only women's participation in the Olympic Movement as athletes, but also their outstanding work in leadership posts within the global structure of the IOC (7). Currently it is possible to highlight that women represent 37.5% of the members of the IOC Assembly (International Olympic Committee, n.d.), however, there is still work to be done to achieve equity both in competitions and in the organization of these and management tasks.

According to the evidence available in the IOC's archives and memoirs, the incorporation of women into the governing boards and decision-making of Olympic sport has been a slow and gradual process, marked by persistent structural and cultural barriers. Miragaya (8) points out that it was not until 1981 that the IOC included a woman among its members for the first time, which reflects the entrenched socio-cultural constructs based on gender stereotypes that have historically limited women's access to leadership posts in the sports field. As the author emphasizes, this systematic exclusion is intrinsically linked to power dynamics that have privileged the male perspective and participation in the sport governance at the international level.

This female under-representation pattern has been replicated in sports federations and other governing boards, evidencing the need to implement affirmative measures to promote greater gender equity in decision-making structures. Although significant progress has been made in recent decades, important gaps persist that require a comprehensive approach from a human-rights orientation and gender perspective (6). The successful actions that have permeated the hegemonic structures are aimed at wage equality, employment discrimination, among others, as well as the accelerated growth of women's professional and semi-professional sports leagues (9). However, the sports field has been plagued by different manifestations of gender inequalities (10) and although there are great advances such as the increase in female participation in sporting events and the increase in support for women athletes, there is still a lack of greater coverage by the media giving space to women's performance and careers in the physical activity field (11).

Despite the advances to promote gender equity and equality between women and men within sport, in Mexico as well as in many countries around the world, there are still blank spaces in which work is needed, such as the scarce investment made in sponsorships for female athletes (7, 12, 13), the great differences in the wages of professional athletes compared to male athletes (14), the marked stereotypes within some sports that do not allow the women participation (15, 16) and even differences in the research conducted from the sport science field (17, 18), and the invisibility in the wording and implementation of generic masculine in public policy, as well as the absence of specific programs with a gender perspective for the sport development in children and adolescents (6).

The relevance of achieving gender equality goals in sport and physical activity is due to the impact that the women's actions participating in sports spaces can have on the promotion of gender equality within society. According to the literature (19), there have been several cases in which successful women athletes have managed to position themselves as role models for young women and adolescents, leading the way for more women's participation not only in this field but also in some others that have historically been dominated by men (16). In the same sense, in a study carried out to know the factors that influence women's participation in sports, university students stated that physical activity and sports is a variable that favorably impacts attitudes and behaviors towards gender equity in these practices (20). In addition, it has also been reported the relationship between sport and the improvement of attitudes such as confidence, security and self-esteem, as well as leadership skills in women (21–23), helping the empowerment and visibility of pro-equity possible actions (24). These studies have contributed the awareness governments and world sports organizations, allowing the creation of agendas and commitments in the short, medium and long term that have generated an increase in women's participation in disciplines, positions and responsibilities that were previously not allowed or created.

For all the above mentioned, sport must be seen as an agent of change and work on the training of future generations at an early age so that they develop an awareness of the importance of people's equity, regardless of their gender (7) and it is essential to promote more inclusive spaces so that all society's citizens have the same rights and opportunities that characterize a fairer humanity (25). This change must take place in the smallest sphere, i.e., from an individual level followed by the most organized structures, such as government, institutions among others, under a bottom-up scheme and thus have a solid base.

The International Olympic Committee has a wide possibility to promote educational programs that strengthen environments of equality, inclusion and far-reaching actions (26), from local efforts, through Olympic Study Centers and National Olympic Committees. Nevertheless, these efforts are often restricted to specific populations or lack widespread promotion, limiting their potential to reach and impact a broader audience.

A possible approach to increasing the promotion of Olympic values and gender equity in sports lies within institutions that train professionals in sports sciences. These institutions provide a valuable platform for fostering inclusion and gender equality through the implementation of actions that integrate these principles into their educational and professional practices. Universities must consider within their training models content that takes up aspects of the Olympic philosophy, which speaks of creating conditions for a healthier and more ethical sports environment (1). The initiative of the International Olympic Academy (IOA) promotes that educational institutions incorporate Olympic themes in their curricula (27). This action strengthens the training of professionals in the sports field with solid knowledge of the benefits of Olympism with a humanistic perspective that aims to build a more inclusive society.

Currently, most of the Mexican universities that offer careers relevant to physical education and sports training do not include topics related to the history and current scenarios of the Olympic Games in their curricula. Studies carried out in countries such as Mexico Munoz-Helu et al. (28) found difference between women (4.58 ± 0.31) and men (3.17 ± 0.66) perception of equity in sport (*p* < .05), and the sport practice level such regional (4.83 ± 0.17), local (4.06 ± 0.88) and non-practice (4.07 ± 1.15) showing a significant differences (*p* < .05) on social perception of women in sport. Also, the influence of sport practice (17.10 ± 3.98) and non-practice (21.00 ± 2.00) over the perception of the values of the Olympic Movement (*p* < .05) were reported. On the other hand, studies with Spanish samples Gómez-Mármol et al. (29, 30) have pointed out the importance of knowing the Olympic Movement values and its philosophy for physical education and sports science professionals, highlighting the possibility of promoting this knowledge with students and athletes through the implementation of the Olympic values, among which gender equality and equity in sport should be specified. Likewise, some authors have noted the relationship between the Olympic Movement and the gender equality development (31–33) and the perception that exists in university youth around both phenomena (28).

In accordance with the above, the study aims to analyze the moderating effect of gender and sports level on the influence of the Olympic Movement's perception on attitudes towards women's participation in sport among students of Physical Education and Sport Sciences. Supported by empirical evidence, the following research hypotheses are proposed in order to respond to the study's objective: (H1) Women will have positive attitudes and greater social support towards women's participation in sport, while perceiving less equity and access to sports practice compared to men; (H2) Those who play sport at the highest level will have greater Olympic Movement knowledge and a higher perception of the Olympic Movement values, significance and threats; (H3) The gender variable will condition the perception's effect on the Olympic Movement and its implications on attitudes towards women's participation in sport; (H4) The sports level variable will condition the participants' effect on the Olympic Movement perception and its implications on attitudes towards women's participation in sport; (H5) The interaction between the gender and sports level variables will condition the participants' effect on the Olympic Movement perception and its implications on attitudes towards women's participation in sport.

## 2 Materials and methods

### 2.1 Participants

The study was carried out using a cross-sectional design with correlational-descriptive scope in a population of Mexican universities students studying physical education, sports and/or exercise science. As this population undergoes training designed for application with children, teenagers, and even adults in the near future, it is crucial to understand their beliefs and attitudes toward gender equality. Additionally, identifying their knowledge and perspectives on Olympic philosophy is essential, as it contributes to their professional development as future advocates for gender equity and the values of sport. By this reason, we employed a non-probabilistic convenience sampling was used in which the participants had to meet the following inclusion criteria: (a) Be students of an undergraduate or postgraduate degree in physical education, sports and/or exercise science (or its equivalent); (b) Study in a Mexican HEI during the period August 2020–July 2021. A total of 15 different HEIs participated in the study, obtaining a sample of 415 subjects (33.5% women and 66.5% men). The socio-demographic data of the subjects are shown in Table 1.

**Table 1:** Study participants’ socio-demographic information.

Variable	Women		Men		All	
	*f*	%	*f*	%	*f*	%
Educational Institution						
Autonomous University of Occident	6	4.3	29	10.5	35	8.4
Autonomous University of Nuevo Leon	16	11.5	26	9.4	42	10.1
University of Colima	17	12.2	24	8.7	41	9.9
Sonora State University	7	5.0	18	6.5	25	6.0
Autonomous University of Baja California	17	12.2	46	16.7	63	15.2
Autonomous University of Ciudad Juarez	5	3.6	12	4.3	17	4.1
Veracruzana University	26	18.7	33	12.0	59	14.2
YMCA University	6	4.3	13	4.7	19	4.6
UNID	3	2.2	8	2.9	11	2.7
Santillana del Mar University	2	1.4	3	1.1	5	1.2
Higher School of Physical Education and Sport	1	0.7	6	2.2	7	1.7
Autonomous University of Guerrero	30	21.6	47	17.0	77	18.6
National Autonomous University of Mexico	1	0.7	3	1.1	4	1.0
National School of Sports Coaches	2	1.4	6	2.2	8	1.9
Tijuana Border Normal School	–	–	2	0.8	2	0.4
Academic degree						
Undergraduate Student	129	92.8	252	91.3	381	91.8
Postgraduate Student	10	7.2	24	8.7	34	8.2
Currently practicing sport level						
International	2	1.4	1	.4	3	0.7
National	17	12.2	22	8.0	39	9.4
Regional	12	8.6	27	9.8	39	9.4
Local	64	46.0	180	65.2	244	58.8
Non-practice	44	31.7	46	16.7	90	21.7

*N* = 415 subjects; *f* = frequency; *%* = percentage.

### 2.2 Instruments

#### 2.2.1 Olympism vision and its educational repercussions questionnaire

Four scales of the Olympism vision and its educational repercussions questionnaire (OVERQ, *Cuestionario sobre la visión del Olimpismo y sus repercusiones educativas* by its spanish name) were used, which aims to assess the Olympic knowledge level of those who answer it, in addition to analyzing the perception about the repercussions that the Olympic Movement has on society (34). The scale consisted of two sections (See Supplementary Material 1), the first is a questionnaire that measures Knowledge about Olympism (KOL) and its history, it consisting of 10 questions (*e.g., Year of the first Olympic Games*) with four response options (only one is correct); the second part consists of 25 items that are answered using a 5-point Likert scale (0 = Not at all agreed .. 4 = Strongly agree), assesses the perception scale faced by the Olympic Movement threats (OMT, *e.g., in your opinion… Lack of fair play as a negative factor for the future of the Olympic Movement*), the values (OMV, *e.g., To what extent do you think winning is the most important aspect of the Olympic Games?*) and the significance (OMS, *e.g., in my opinion… The Olympic Movement is a humanistic ideal*) that the Olympic Games convey. For the interpretation of the second section of the questionnaire, the sum of the items corresponding to each scale is analyzed, with the possible score ranges for the OMT factor from 0 to 36, 0 to 24 for OMV and 0 to 40 in the case of OMS. The higher the perceived score, the more identified one is with what the scale intends to reflect (34).

Previous research has reported adequate internal consistency values for the different questionnaire scales (29, 30, 34, 35). In our study, the scales' reliability indicators reached the following McDondald's Omega and Cronbach's alpha coefficients, *ω* = 0.72 and *ɑ* = 0.70 for the KOL questionnaire, *ω* = 0.71 and *ɑ* = 0.74 for the OMT scale, *ω* = 0.69 and *ɑ* = 0.65 in the case of the OMV scale, as well as *ω* = 0.82 and ɑ = 0.82 in the OMS scale. It is possible to observe that the instrument's reliability coefficients are mostly above the cut-off point considered appropriate (≥.70), however, it has already been pointed out by some researchers that even alpha values above 0.60 can be regarded as not affecting the overall scale (36, 37).

#### 2.2.2 Scale of attitudes toward women's participation in sports

To measure students' perceptions of attitudes towards women's participation in sport, the Scale of Attitudes Toward Women's Participation in Sports was used (SATWPS) (38), which was modified and validated based on the Perception Survey of the Relationship Women and Sport, designed by the National Women's Institute (39). The attitudes scale that the subjects have towards the women's participation in sports activities is composed of 14 items with a Likert-type response where 1 is equivalent to “totally disagree” and 5 to “totally agree”. The total of items is divided into three factors (See Supplementary Material 2): (1) Perception of women in sport (PWS, *e.g.,* “*A woman can go as far as a man if she sets her mind to it”*); (2) Perception of equity in sport (PES, *e.g., “Male and female athletes receive the same support from sports institutions”*); (3) Social Support for women in sports (SSWS, *e.g., “An equal number of sports scholarships are offered to men and women”*). The scale is rated by each of the factors, averaging the items that make up each of them. The closer to five is the score obtained, the greater identified is the subject's attitude perception described in each one (38).

The scale has previously been used in different contexts, reporting Cronbach's alpha coefficients greater than .70 both on its overall scale and on most of the four factors (20, 38, 40, 41). In the present study, values of McDondald's Omega and Cronbach alpha of *ω* = 0.83 and *ɑ* = 0.82 for the PWS factor, *ω* = 0.82 and *ɑ* = 0.82 for the PES factor, while for the SSWS were *ω* = 0.81 and *ɑ* = 0.80. Regarding the overall scale, a reliability of *ω* = 0.83 and ɑ = 0.83 was obtained. The reliability data presented are deemed adequate since they are above 0.60 considered as an acceptable limit value for psychometric scales (36, 37).

### 2.3 Procedure

To carry out the study, the online version of both questionnaires was transcribed through the Google Forms application, taking care not to modify each of the items and their respective response. After this process was completed, the academic coordinators of the educational programs were contacted so that they could support the application of the measurement instruments. The research objectives were established, as well as the questionnaires collection mechanism. Subsequently, the link to the form was shared with the institutions through a mobile messaging application (WhatsApp). During the process of answering the instruments, the research group monitored their progress and kept abreast to any doubts through the same messaging platform. The participants answered the questionnaires voluntarily, agreeing to participate in the study. For this research, the guidelines and ethical recommendations for the subjects' handling and the data obtained were followed as outlined in the Helsinki declaration (42), following the specific standards for sports medicine and exercise sciences research (43).

### 2.4 Statistical analyses

Reliability of the instruments was first tested for data analysis using Cronbach's alpha coefficient. Subsequently, frequencies and percentages of the socio-demographic data describing the sample were identified, as well as the correct and incorrect answers for the Olympic knowledge questionnaire. In addition, the behavioral description of the data was carried out by obtaining the mean (M) and standard deviations (SD) central tendency measures for the scales and factors of both questionnaires. To allow an appropriate application of the contrast statistics between the study groups, the following variables were coded: gender (woman = −0.50; man = 0.50), sports practice level (not practiced = 0; local = 1; regional = 2; national = 3; international = 4). Analyses of variance were then performed according to gender using the student's t-test, while for comparisons by sports practice level, an ANOVA with Hochberg's *post hoc* GT2 was applied (due to the difference in the sample size per group). The *p*-value <.05 was considered as a significant cut-off point for all analyses.

To comply with the study's main objective, an independent double moderation analysis (Model 2) was executed using the PROCESS V.3.5 macro component for SPSS V.25 (44). The moderating effect that the sports practice (level) and gender variables have on the relationship between the OVERQ scales, and the factors measured by the SATWPS was observed. By applying the resampling technique to 10,000 samples, the three model effects for the level (b1, b2 and b3) were determined with confidence intervals at a 95% rate. Using the pick-a-point approach, three points for the practice level were generated, which can be considered as non-practice, low-moderate level (local and regional) and high level (national and international), while the gender variable is considered dichotomous.

## 3 Results

Knowledge about Olympism of the participants (Table 2) reflected that more than 80% know about the Olympic Movement historical events such as the city where the first modern Olympic Games were held, the city where the Olympic Games were held in 2016 and the meaning of the Olympic rings. On the contrary, only one respondent, who pronounced the phrase “the important thing is not to win, but to participate” in the 1908 Olympic Games of London, had an error percentage of over 90%. Regarding the score obtained in Olympism knowledge according to gender and sports practice level (Table 3), it stands out that women on average have significantly greater knowledge (*p* < .05) than men, as well as those who compete at the international level achieved a better score (*p* > .05) in KOL regard to the others.

**Table 2:** Participants’ olympism knowledge.

No.	Question	*f*	%
1	¿Which city held the first modern Olympic Games?		
	Correct	338	81.4
	Incorrect	77	18.6
2	¿In what year were the first modern Olympic Games held?		
	Correct	273	65.8
	Incorrect	142	34.2
3	¿Which city hosted the 2016 Olympics?		
	Correct	338	81.4
	Incorrect	77	18.6
4	¿Where were the 2008 Olympics held?		
	Correct	269	64.8
	Incorrect	146	35.2
5	¿Who pronounced the phrase “the important thing is not to win, but to participate” in the1908 Olympic Games of London?		
	Correct	39	9.4
	Incorrect	376	90.6
6	The Olympic Charter is..		
	Correct	258	62.2
	Incorrect	157	37.8
7	¿Who was the restorer of the modern Olympic Games?		
	Correct	298	71.8
	Incorrect	117	28.2
8	¿What does the Olympic symbol of the five interlocking rings represent?		
	Correct	393	94.7
	Incorrect	22	5.3
9	Who is currently the President of the International Olympic Committee (IOC)?		
	Correct	269	64.8
	Incorrect	146	35.2
10	What is the Olympic motto?		
	Correct	244	58.8
	Incorrect	171	41.2

*n* = 394 subjects; *f* = frequency; *%* = percentage.

**Table 3:** Analysis of variances on olympism and attitudes towards women's participation in sport variables.

Compared groups	KOL	OMT	OMV	OMS	PWS	PES	SSWS
Gender							
Woman	6.97 ± 2.20	19.85 ± 6.95	17.51 ± 3.54	25.97 ± 5.22	4.60 ± 0.65	2.98 ± 0.83	2.64 ± 0.88
Man	6.34 ± 2.17	19.80 ± 6.70	17.33 ± 3.61	25.14 ± 5.12	4.45 ± 0.67	3.36 ± 0.88	2.97 ± 0.90
*t*	2.78**	0.06	0.50	1.55	2.26*	−4.23***	−3.64**
Practice level							
International	7.00 ± 3.46	16.00 ± 7.00	14.67 ± 4.04	25.00 ± 3.46	4.00 ± 1.32	2.60 ± 0.72	2.67 ± 0.33
National	6.49 ± 2.39	20.69 ± 6.90	17.72 ± 3.95	27.69 ± 4.69	4.57 ± 0.65	3.33 ± 0.90	2.96 ± 1.05
Regional	6.23 ± 2.64	18.64 ± 7.40	17.31 ± 3.85	25.28 ± 4.99	4.56 ± 0.64	3.23 ± 0.86	2.99 ± 1.04
Local	6.52 ± 2.06	20.11 ± 7.11	17.36 ± 3.67	25.49 ± 5.18	4.49 ± 0.66	3.29 ± 0.91	2.86 ± 0.92
Doesn't practice	6.79 ± 2.27	19.28 ± 5.38	17.43 ± 3.02	24.32 *±* 5.21^a^	4.49 ± 0.70	3.07 ± 0.79	2.78 ± 0.76
*F*	0.52	0.95	0.53	2.98*	0.64	1.56	0.52

KOL, knowledge about Olympism; OMT, Olympic movement threats; OMV, Olympic movement values; OMS, Olympic movement significance; PWS, perception of women in sport; PES, perception of equity in sport; SSWS, social support for women in sport.

^a^ Difference with national level.

* *p* < .05; **p < .01.

With respect to the participants' perception of the threats, values, and meaning reflected in the Olympic Movement (Table 3), only in the OMS was observed a significant difference according sports practice level (F = 2.98, *p* < .05).

**Figure 1:**
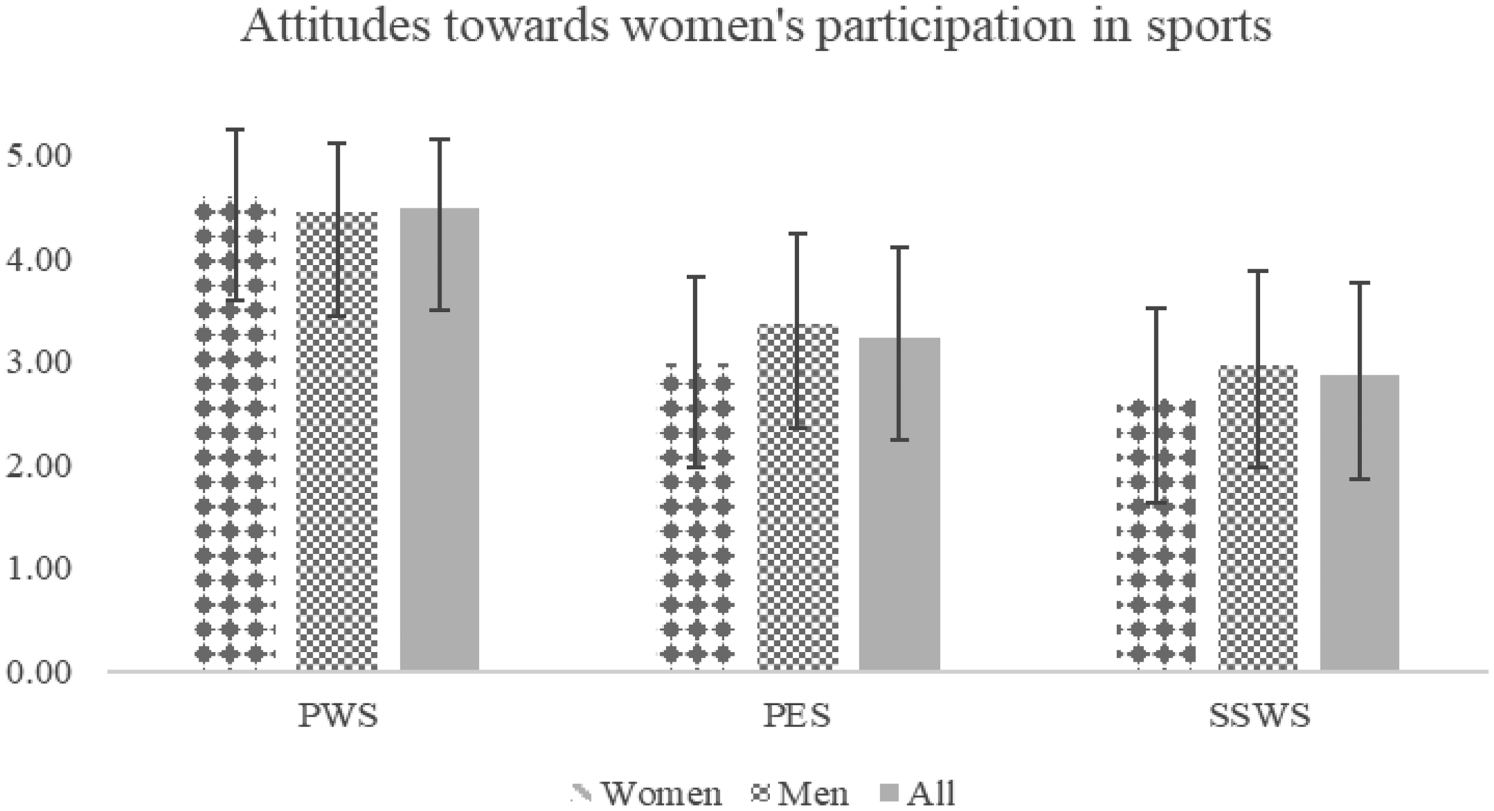
Mean and SDs on the perception of attitudes towards women's participation in sport. 1 = totally disagree; 2 = disagree; 3 = undecided; 4 = agreed; 5 = totally agree. (PWS *α* = .83; PES *α* = .82; SSWS *α* = 76; Global scale *α* = .79).

In reference to the perception of women's participation in sport (Figure 1), the results of this study show a positive perception of women in sport (M = 4.50 ± 0.67), however, the constructs of perception of equity in sport (M = 3.13 ± 0.84), and social support for women in sport (M = 3.27 ± 0.52) are observed just above the possible mean value.

Likewise, when the differences according to gender and sports practice level were analyzed (Table 3), it is identified that women reflect higher scores than men with respect to the PWS (*t* = 2.26, *p* < .05), while they indicate a lower PES (*t* = −4.23, *p* < .01) and SSWS (*t* = 3.74, *p* < .01) compared to what was exposed by men. On the other hand, in the sports practice level analysis, no significant differences (*p* < .05) were observed in the variables related to the perception of women's participation in sport.

**Figure 2:**
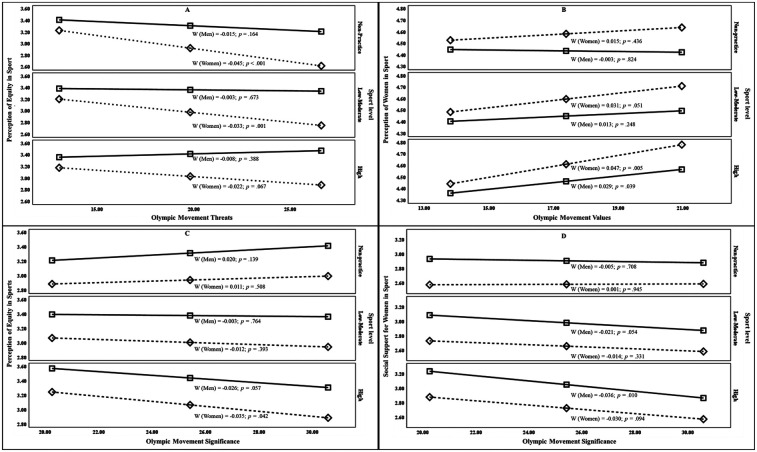
Moderating effect of gender and sports practice level on the influence on the perception of the Olympic movement and gender equity in sport in students of physical education and sports sciences. **(A)** Gender and sport practice-level conditional effects over Olympic movement threats interaction with perception of equity in sport; **(B)** Gender and sport practice-level conditional effects over Olympic movement values interaction with Perception of women in sport; **(C)** Gender and sport practice-level conditional effects over Olympic movement significance interaction with Perception of equity in sport; **(D)** Gender and sport practice-level conditional effects over Olympic movement significance interaction with Social support for women in sport; Non-practice: Does not play sports; Low-moderate: Local and regional sports practice level; High: National and international sports practice level.

Finally, Table 4 and Figure 2 show the results of the double moderation analysis that are statistically significant, in which the moderation exercised by the variables gender (W) and sports level (Z) on the effect of the independent variables (X) KOL, OMT, OMV and OMS of the OVERQ on the dependent variables (Y) PWS, PES, and SSWS of the SATWPS is observed.

**Table 4:** Moderating conditional effects for each significant model.

Model 1	Equity perception in sport (Y)		
	Effect	Coefficient (SD)	95% CI
(X) Olympic movement threats	β1 →	−0.033 (0.011) *p* = .003	−0.0548, −0.0110
(W) Gender	β2 →	−0.213 (0.273) *p* = .435	−0.7494, 0.3235
X * W	β3 →	0.030 (0.013) *p* = .020*	0.0047, 0.0558
(Z) Sports level	β4 →	−0.204 (0.157) *p* = .194	−0.5119, 0.1046
X*Z	β5 →	0.013 (0.008) *p* = .079^+^	−0.0016, 0.0283
Constant	*^iM^*→	3.755 (0.229) *p* *<* .001*	3.3069, 4.2024
	*R^2^* = 0.076; *F* (5, 388) = 6.421, *p* < .001		
Unconditional interaction X * W * Z	*R^2^* = 0.019; *F* (2, 409) = 4.255, *p* = .014*		
Model 2	Perception of women in sport (*Y*)		
	Effect	Coefficient (SD)	95% CI
(X) Olympic movement values	β1 →	−0.002 (0.016) *p* = .904	−0.0299, 0.0338
(W) Gender	β2 →	0.176 (0.346) *p* = .610	−0.5040, 0.8569
X * W	β3 →	−0.019 (0.020) *p* = .338	−0.0569, 0.0196
(Z) Sports level	β4 →	−0.306 (0.185) *p* = .099^+^	−0.6694, 0.0581
X*Z	β5 →	−0.019 (0.011) *p* = .077^+^	−0.0021, 0.0391
Constant	*^iM^*→	4.4714 (0.286) *p* *<* .001*	3.9085, 5.0343
	*R^2^* = 0.054; *F* (5, 388) = 4.412, *p* < .001		
Unconditional interaction X * W * Z	*R^2^* = 0.011; *F* (2, 409) = 2.249, *p* = .106^+^		
Model 3	Equity perception in sport (Y)		
	Effect	Coefficient (SD)	95% CI
(X) Olympic movement significance	β1 →	0.021 (0.013) *p* = .119	−0.0055, 0.0472
(W) Gender	β2 →	−0.146 (0.453) *p* = .747	−0.7445, 1.0358
X * W	β3 →	0.009 (0.017) *p* = .605	−0.0250, 0.0429
(Z) Sports Level	β4 →	0.736 (0.264) *p* = .005*	0.2176, 1.2534
X*Z	β5 →	−0.026 (0.010) *p* = .008*	−0.0456, −0.0066
Constant	*^iM^*→	2.253 (0.345) *p* *<* .001*	1.9048, 3.2607
	*R^2^* = 0.065; *F* (5, 388) = 5.432, *p* < .001		
Unconditional interaction X * W * Z	*R^2^* = 0.016; *F* (2, 409) = 3.550, *p* = .029*		
Model 4	Social support for women in sport (Y)		
	Effect	Coefficient (SD)	95% CI
(X) Olympic movement significance	β1 →	0.002 (0.014) *p* = .887	−0.0254, 0.0293
(W) Gender	β2 →	0.484 (0.470) *p* = .303	−0.4402, 1.4079
X * W	β3 →	−0.006 (0.018) *p* = .727	−0.0415, 0.0290
(Z) Sports Level	β4 →	0.533 (0.274) *p* = .052^+^	−0.0050, 1.0702
X*Z	β5 →	−0.018 (0.010) *p* = .085^+^	−0.0380, 0.0025
Constant	*^iM^*→	2.679 (0.358) *p* *<* .001*	1.9755, 3.3830
	*R^2^* = 0.046; *F* (5,388) = 3.765, *p* = .002*		
Unconditional interaction X * W * Z	R^2^* = 0.007; *F* (2, 409) = 1.576, *p* = .208		

* Statistically significant (*p* < .05); + = statistically marginal (*p* < .10).

## 4 Discussion

The present study objective was to analyze the conditional role that gender and sports level have over the influence that the perception of the Olympic Movement has on attitudes towards women's participation in sport in a group of Physical Education and Sports Science students. According to the literature review carried out, this is the first study that aims to explain the influence that these factors can generate on the relationship between the aforementioned variables. The main finding of our research obtained through the double moderation analysis which allows us to observe that in the students observed, both gender and sports practice level can moderate the effect that the Olympic Movement perception of the threats, values and significance has on the perception of women participation in sports, equity and social support for women's athletes. To enhance clarity, the discussion will be organized and presented under the following sub-themes.

### 4.1 Sports practice as a generator of more realistic perspectives on gender equity in sport

Among students from not-practice sports group, being female has been shown to influence the perceived impact of threats to the Olympic Movement on gender equity. For women, the greater the perception of threats to the future of the Olympic Movement, the lower the identified level of gender equity in sports. This can be attributed to the widespread societal belief that Olympism operates within a framework that promotes gender equity values. However, when actions that undermine the role of athletes in sporting events are brought to light, a perceived disconnect emerges between the Olympic Movement's actions and its stated gender equity principles (45).

Similarly, for individuals participating in low- to moderate-level sports, gender plays a significant moderating role in shaping perceptions of gender equity in sports. This effect is significant among women, where perceived threats to the Olympic Movement intensifies concerns about gender equity. In contrast, no significant gender-based effect is observed for men. Perceptions of inequity are largely influenced by the unequal distribution of resources and media attention to women's sports (38). This sense of inequity becomes evident when comparing female athletes with their male counterparts in terms of income, recognition, and opportunities. Male athletes generally enjoy greater funding and attract investment from various sponsors, thereby ensuring a longer-lasting professional career (46). Such disparities can negatively affect the motivation and well-being of female athletes. In this context, the Olympic Movement plays a crucial role in shaping perceptions of equity among female athletes (47).

This is supported by the findings among participants who compete in high-level sports, where no significant gender-based moderation was observed in their perception of how threats to the Olympic movement impact gender equity. High-level male and female athletes are often more attuned to historical, economic, and media-related aspects (48), enabling them to recognize greater gender inequities compared to athletes participating in local tournaments.

In contrast, among study participants who do not-practice sports, gender did not significantly moderate the effect of perceived OMV on the perception of PWS (i.e., the belief that women have the same right to engage in sports as men). Individuals who do not practice sports tend to attribute women's participation in sports more to the values promoted by the Olympic Movement (49) rather than to the actions of inequality observed within the sports environment.

Expanding on the influence of OMV on perceptions of PWS, the present study found that being a woman who practices sports—whether at low-moderate or high levels—significantly influenced the interaction between OMV and perceptions of women's involvement in sports. Women athletes tended to have a more positive outlook, believing that the values promoted by the Olympic Movement enhance the perception of women's participation in sports practices. Consistent with (50), the IOC has undertaken specific actions to increase women's participation in the Olympic Games and sports. These efforts include initiatives led by Olympic stakeholders and the implementation of the Olympic Values Education Program (26). This connection between OMV and the perception PWS has been reported as positive (51) and particularly evident in specific cases, such as skateboarding and surfing (33).

Regarding the influence of OMS on PES, men (particularly those who do not engage in sports or practice at low to moderate levels) tend to view the Olympic Movement as a space largely free of gender inequality. In contrast, elite women athletes perceive a stronger association between gender inequality and a positive view of OMS. This disparity can be explained by the lived experiences of women athletes, who often encounter gender-based challenges throughout their sports careers, reinforcing their perception of inequalities in sports practices (52). A possible explanation for this phenomenon is that women may hold a deeper conviction about the importance of Olympic significance, which heightens their awareness of systemic barriers to accessing sports. This includes fewer opportunities and resources available to women, even at the elite level (18). In this regard, studies by Talleu (53), Biernat et al. (54), De Oliveira et al. (50), and Whelan (55) suggest that individuals with a critical perspective on sports institutions are more likely to interpret these systemic challenges as evidence of an inadequate or biased system (one that disproportionately disadvantages certain groups), particularly women.

On the other hand, for men who do not practice sports and for women across all levels of sports participation, our results showed no significant relationship between OMS and SSWS. This suggests an assumption that the values and principles of the Olympic Movement inherently promote equal participation between women and men (51). However, for men who are low-moderate and high-level athletes, a more positive perception of OMS is associated with a recognition of inequality practices in sports (56). This indicates that, for these groups, a deeper engagement with or understanding of the Olympic Movement's ideals may reveal discrepancies between its principles and the reality of gender equity in sports.

### 4.2 Differences between women and men sports science students in the vision of gender equality and the Olympic movement

Regarding the scores obtained in KOL by gender and sports practice level (Table 3), it is notable that, on average, women demonstrated significantly greater knowledge (*p* < .05) compared to men. According to Gómez-Mármol et al. (30), the level of knowledge is often associated with age; however, the growing interest in the Olympic Games in recent years has become a key factor driving the desire to acquire more information on this topic (57). Furthermore, as previously highlighted, women may establish a stronger connection between Olympic ideals and universal values (51), which could contribute to their higher levels of KOL.

With respect to the participants' perceptions related to OMT, OMV, and OMS (Table 3), substantial differences were observed only in the OMS based on the level of sports practice (F = 2.98, *p* < .05). This finding indicates that engaging in sports is a key factor in recognizing the OMS, as compared to those who do not participate in sports (58).

In reference to the perception of PWS (Figure 1), the results of this study reveal a generally positive perception (M = 4.50 ± 0.67). However, the constructs of PES (M = 3.23 ± 0.88) and SSWS (M = 2.86 ± 0.91) are observed to be just above the potential mean value, indicating room for improvement. When differences based on gender and sports practice level were analyzed (Table 3), women reported significantly higher scores than men in their perception of women in sports (t = 2.26, *p* < .05). However, women reported significantly lower perceptions of equity in sports (t = −4.23, *p* < .001) and social support for women in sports (t = −3.64, *p* > .01) compared to men. These findings align with previous research, which highlights that women's sports are conducted under conditions of inequality compared to men's sports (7, 52).

These results underscore the urgent need to develop policies not only aimed at promoting the acceptance of women in sports but also at facilitating their access and reducing the persistent inequality gap. Efforts should focus on addressing systemic disparities to ensure a more equitable environment for women in sports (7, 10, 12). On the other hand, it is important to generate campaigns to raise awareness about the importance of women's participation in sports, through educational programs for close groups of athletes such as family members and community of influences with the purpose of highlighting the benefits related to the practice of physical activity, self-esteem and empowerment of women through sports (59), promoting public policies that promote sports initiation in women from an early age in environments of equality.

Furthermore, this study shows results from a specific population that are students of exercise science or related careers. It is possible to observe that in eight of the 10 questions that evaluate the Olympic Movement knowledge, more than 60% of the participants answered correctly, which according to the literature is related to their academic training, due to the influence that Olympism exerts on the training areas related to physical activity and sport (60). For this reason, universities must seek extracurricular strategies within their models to disseminate Olympic education, either through sports and cultural programs, or generate spaces such as educational centers related to Olympism topics that are available to students and support their training, since this philosophy uses some disciplines and sciences to achieve its goals (61).

Sports professionals must have knowledge, competencies and skills that promote sustainability in sport (62), in addition to promoting fundamental values ​​such as inclusion and gender equality by incorporating the Olympic ideals into their academic training, considering that the dimensions of Olympic education are closely related to the premises of comprehensive educational models, whose purpose is to train individuals who practice universal values ​​as a lifestyle, thus promoting the harmonious development of their intellectual, physical and aesthetic capacities (63) the importance of incorporating programs based on Olympism is confirmed.

## 5 Recommendations

Without a doubt, the findings of this study reveal that Olympic knowledge and values ​​influence the perception of gender equality, which allows us to intuit the important implications of the design and implementation of Olympic education programs in the contents of basic education, sports instruction, and, of course, in degree programs aimed at training physical education teachers. Curricular or instructional design must consider an intersectional approach, considering that gender coexists with variables such as identity, socioeconomic status, ethnicity, and geographic location. Considering intersectionality is educationally possible to address the complex and multifaceted barriers that women face in sports participation, as highlighted by Ricardo et al. (64), women face obstacles to physical activity from childhood, including body insecurity and concerns about social acceptance, which often result in resistance to sports participation. Therefore, promoting modern social norms that create supportive environments is essential to encouraging greater female involvement in sports.

Likewise, the identification in the study of lower perceptions of equity and social support for women among the female participants themselves highlights a structural inequality that seems to have no name, as Betty Friedan indicates in Wood (65) “women are incapable of interpreting their own situation of inequality” (p.114). From a structural perspective, female subordination is explained by the lack of guarantees of equality at the legislative, political, work, emotional and family levels (66). This clearly makes evident in the case of female athletes, the absence of gender policies and sports policies that offer strategies and solutions to dismantle structural inequality in the sports system.

Various theoretical positions on the social construction of gender allow us to affirm that a partial or fragmented strategy (political or educational) fails to generate profound changes in the daily lives of athletes, much less reduce the barriers and inequalities existing in the sports system. Etchezahar (67) affirms that we must influence the change in expectations about the feminine and masculine, the principles, norms, and cultural representations about the role of the athlete. For their part, Hopkins et al. (68) mention that there are key factors to promote women's participation in sports at early ages, highlighting the family environment, biological, and socioeconomic factors. Therefore, it is recommended to consider community interventions as strategic actions to expand the field of action and begin to mainstream the gender perspective in various social structures (family, friends, or sports teams). These community structures can be sports clubs, recreational facilities, sports centers, community organizations, sports associations, and the media.

## 6 Limitations

A methodological limitation of the present study is the integration of the sample, since women represent only 33.5% of the participants compared to 66.5% of men. Being research with a gender perspective, this imbalance of the sample represents an inconsistency and incongruence to the principles of equity and methodologically to the validity of the findings with respect to gender differences. However, this limitation also refers to the underrepresentation of women in the field of physical education and sports sciences where historically enrollment has shown gender disparities in these careers. This can be verified in the study by Matus et al. (69), which shows how from 2005 to 2019 in various countries the enrollment of women in careers is 31.1%.

In the same sense, the sample is also made up only of university students from physical education and sports programs, which limits the generalization of the findings to broader populations in the educational and university context. However, scientific evidence from the educational field makes it possible to reduce gender gaps through quality education (70). Therefore, beginning to explore these issues in the university population that is trained in the field of physical education and sports, and that in the immediate future will be the reproducer of stereotypes or the one who promotes proactive attitudes towards gender equality, potentially provides a more progressive perspective than what could exist in other populations or university courses. Finally, a limitation of the study was the lack of socio-economic information and the absence of participants' perceptions of government policies on gender equity in sports, which restricted the depth of the analysis.

## 7 Conclusion

Having literacy on physical culture, feminist pedagogies, and therefore, Olympism will allow athletes, students in training in applied sports sciences and citizens to generate a critical awareness based on human rights and sports values against the increasingly obsolete stereotyped narratives, normatives or cultural procedures of the world's sports systems and organizations. Currently, there are already good practices that exemplify the strengthening of institutions by incorporating women in an equally manner, which is confirmed in the positive perception of women's participation in sport observed in this study, as well as the influence on the equity perception and the role that sports institutions can have, being mainly women and athletes of highest competitive level who consider that the system is inadequate or unfavorable for certain groups, especially for women.

Therefore, higher education institutions dedicated to training sports professionals must play a more active role in implementing curricula that promote the principles of Olympism and contribute to breaking down historical barriers. This approach is essential to fostering a more inclusive sports environment where universal values and respect for human rights are prioritized.

## Data Availability

The raw data supporting the conclusions of this article will be made available by the authors, without undue reservation.

## References

[1] Villegas Estrada CE. La agenda olímpica 2020: desafíos y oportunidades de las 40 recomendaciones para la sostenibilidad y credibilidad del movimiento olímpico [The Olympic agenda 2020: challenges and opportunities of the 40 recommendations for the sustainability and credibility of the Olympic movement]. Citius Altius Fortius. (2016) 9:1–16. 10.15366/citius2016.9.2.001

[2] International Olympic Committee. Luchando por la igualdad de género [Fighting for gender equality] (2021) Available at: https://olympics.com/athlete365/es/articles/integrity/luchando-por-la-igualdad-de-genero (Accessed June 17, 2023).

[3] Chatziefstathiou D. Pierre de coubertin y los derechos humanos: deporte como derecho humano [Pierre de coubertin and human rights: sport as human right]. Citius Altius Fortius. (2020) 13:15–22. 10.15366/citius2020.13.2.003

[4] Sagarzazu Olaizola I Lallana del Rio I. Estrategias del comité olímpico internacional para la igualdad de género en el deporte y la imagen mediática de las deportistas. [Strategies of the international Olympic committee for gender equality in sport and the Media image of female athletes]. In: Suárez Villegas JC Liberia Vayá I Zurbano Berenguer B, editors. I Congreso Internacional de Comunicación y Género. Seville: University of Seville (2012). p. 2048–68.

[5] Morano M Robazza C Ruiz MC Cataldi S Fischetti F Bortoli L. Gender-Typed sport practice, physical self-perceptions, and performance-related emotions in adolescent girls. Sustainability. (2020) 12:8518. 10.3390/su12208518

[6] Salazar CM. Mujer y deporte: comportamiento de la política pública mexicana de 2000–2022. CienciaUAT. (2023) 18:141–57. 10.29059/cienciauat.v18i1.1738

[7] Flores Fernandez Z. Mujer y deporte en méxico. Hacia una igualdad sustancial. [Women and sport in México. Towards a substantive equality]. Retos. (2019) 37:222–6. 10.47197/retos.v37i37.71684

[8] Miragaya AM. The process of inclusion of women in the Olympic Games (doctoral thesis). Universidad Gama Filho, Rio de Janeiro (2006). https://library.olympics.com/Default/doc/SYRACUSE/26494/the-process-of-inclusion-of-women-in-the-olympic-games-by-ana-maria-miragaya-lamartine-da-costa-supe?_lg=en-GB

[9] Pegoraro A Taylor T. Editorial: women’s professional sport: understanding distinctiveness. Front Sports Act Living. (2021) 3:e806247. 10.3389/fspor.2021.806247 PMC8702796 34957400

[10] Meier HE Konjer MV Krieger J. Women in international elite athletics: gender (in)equality and national participation. Front Sports Act Living. (2021) 3:e709640. 10.3389/fspor.2021.709640 PMC8429847 34514387

[11] Salido Fernández J. Olimpismo, género y comunicación: una aproximación al deporte femenino y a su representación en los medios deportivos [Olympism, gender and communication: an approach to women’s sport and its representation in sports media]. Comunicación y Género. (2020) 3:173–82. 10.5209/cgen.68559

[12] Mendoza-Farias FJJ Quintal-López RI Paredes LJ. Análisis de la incorporación de la perspectiva de género en la política pública deportiva en méxico: el caso de la comisión nacional de cultura física y deporte [Analysis of the incorporation of the gender perspective in public sports policy in Mexico: the case of the national commission of physical culture and sports]. Polít Glob Ciudadanía. (2019) 5:75–89. 10.29105/pgc5.9-3

[13] Rasmussen K Dufur MJ Cope MR Pierce H. Gender marginalization in sports participation through advertising: the case of Nike. Int J Environ Res Public Health. (2021) 18:7759. 10.3390/ijerph18157759 34360052 PMC8345737

[14] Flores Fernandez Z Chávez Bermudez BF Mier Cisneros R Obregón Avelar KA. Violencia de género en el deporte [Gender violence in sport]. Retos. (2022) 43:808–17. 10.47197/retos.v43i0.85842

[15] Hermann JM Vollmeyer R. “Girls should cook, rather than kick!” – female soccer players under stereotype threat. Psychol Sport Exerc. (2016) 26:94–101. 10.1016/j.psychsport.2016.06.010

[16] Howe HS Welsh TN Sabiston CM. The association between gender role stereotypes, resistance training motivation, and participation. Psychol Sport Exerc. (2017) 33:123–30. 10.1016/j.psychsport.2017.08.006

[17] Fraser KK Kochanek J. What place does elite sport have for women? A scoping review of constraints. Front Sports Act Living. (2023) 5:e1121676. 10.3389/fspor.2023.1121676 PMC10300643 37389274

[18] Martínez-Rosales E Hernández-Martínez A Sola-Rodríguez S Esteban-Cornejo I Soriano-Maldonado A. Representation of women in sport sciences research, publications, and editorial leadership positions: are we moving forward? J Sci Med Sport. (2021) 24:1093–7. 10.1016/j.jsams.2021.04.010 34024735

[19] McCann M. El fútbol femenino: los implicaciones de ser una futbolista femenina en una cultura machista [Women’s football: the implications of being a female footballer in a macho culture]. Independent Study Project (ISP) Collection. 2322. (2016). Available at: https://digitalcollections.sit.edu/isp_collection/2322/ (Accessed May 23, 2023).

[20] Cruz-Morales KN Muñoz-Helú H Rios Mena Gaxiola JA Guimarães-Mataruna AF Mataruna-Dos-Santos LJ Reynoso-Sánchez LF. Factores que influyen sobre las actitudes hacia la participación de la mujer en el deporte [Factors influencing attitudes towards women’s participation in sport]. Sportis. (2022) 8:396–415. 10.17979/sportis.2022.8.3.9077

[21] Meier M. The value of female sporting role models. Sport Soc. (2015) 18:968–82. 10.1080/17430437.2014.997581

[22] Navarro Domínguez B Cerrada Nogales JA Abad Robles MT Giménez Fuentes-Guerra FJ. El desarrollo del respeto en la formación deportiva: una revisión sistemática [The development of respect in sports training: a systematic review]. Sportis. (2020) 6:533–54. 10.17979/sportis.2020.6.3.6527

[23] Vallejo AG Alguacil Jiménez M. Influencia de la actividad físico-deportiva en el rendimiento académico, la autoestima y el autoconcepto de las adolescentes: el caso de la isla de tenerife [Influence of physical-sports activity on academic performance, self-esteem and self-concept of adolescent girls: the case of the island of tenerife]. Retos. (2022) 46:120–8. 10.47197/retos.v46.93496

[24] Cooky C. Sociology of gender and sport. In: Risman B Froyum C Scarborough W, editors. Handbook of the Sociology of Gender. Handbooks of Sociology and Social Research. Chicago, Ilinois: Springer (2018). p. 459–67 10.1007/978-3-319-76333-0_33

[25] Casey M Doherty A Elliott SK Norman L. Editorial: engaging women and girls in community sport: building an equitable and inclusive future. Front Sports Act Living. (2022) 4:947626. 10.3389/fspor.2022.947626 35769221 PMC9236571

[26] Theodorakis Y Georgiadis K Hassandra M. Evolution of the Olympic movement: adapting to contemporary global challenges. Soc Sci. (2024) 13:326. 10.3390/socsci13070326

[27] Buhajezuk J. Propuestas y recomendaciones para impulsar el rol que el movimiento olímpico puede desempeñar para promover los derechos humanos en la actualidad [Proposals and recommendations to advance the role that the Olympic movement can play in promoting human rights today]. Citius Altius Fortius. (2020) 13:73–7. 10.15366/citius2020.13.1.005

[28] Munoz-Helu H Reynoso-Sánchez LF Noelia Cruz-Morales K Korinna Zazueta-Beltran D Abril Morales-Beltran R Garcia-Flores JJ. Gender and sports practice are related to the perception of the Olympic movement and gender equity in sport. Int J Sport Stud Health. (2022) 4:e126630. 10.5812/intjssh-126630

[29] Gómez-Mármol A Sánchez-Alcaraz BJ Bazaco JM Molina JM. La percepción del olimpismo de los estudiantes universitarios de educación física y ciencias del deporte: un estudio en la comunidad autónoma de murcia [The olympism perception of university students of physical education and sports sciences: a study in the autonomous community of murcia]. J Sport Health Res. (2015) 7:103–12. https://www.researchgate.net/publication/277475524

[30] Gómez-Mármol A Calabuig Martí V Bazaco Belmonte MJ. Conocimientos sobre olimpismo, sus riesgos y valores entre los docentes de educación física en secundaria en función del género [Olympism knowledge, its risks and values among secondary school physical education teachers according to gender]. Acciónmotriz. (2018) 21(1):15–21.

[31] Koenigsberger A A. Gender equality in the Olympic movement: not a simple question, not a simple answer. J Philos Sport. (2017) 44:329–41. 10.1080/00948705.2017.1359616

[32] Teetzel S. Rules and reform: eligibility, gender differences, and the Olympic games. Sport Soc. (2011) 14:386–98. 10.1080/17430437.2011.557275

[33] Wheaton B Thorpe H. Action sports, the Olympic games, and the opportunities and challenges for gender equity: the cases of surfing and skateboarding. J Sport Soc Issues. (2018) 42:315–42. 10.1177/0193723518781230

[34] Molina JM. Visión del Olimpismo y sus Repercusiones Educativas entre Universitarios de Magisterio de Educación Física, Grado en Educación Primaria y Grado en Ciencias de la Actividad Física y el Deporte de la Región de Murcia [Olympism Vision and its Educational Repercussions among University Teachers of Physical Education, Primary Education Degree and Physical Activity and Sports Sciences Degree of the Region of Murcia] (tesis doctoral). Universidad Católica de San Antonio, Murcia (2011).

[35] Gómez-Mármol A Sánchez-Alcaraz Martínez B Molina Morote J Bazaco Belmonte MJ. Estudio preliminar para el diseño y validación del “cuestionario sobre la visión del olimpismo y sus repercusiones educativas (CUVOREDU)” preliminary study for the design and validation of the “olympism vision and its educational repercussions questionnaire (OVERQ)]. Rev Estud Exp Educ. (2016) 15:129–44. 10.21703/rexe.2016281291447

[36] Hair JF Black WC Babin BJ Anderson RE. Multivariate Data Analysis. 7th ed New York: Pearson Prentice Hall (2009).

[37] Nunnally J Bernstein I. Teoría Psicométrica [Psychometric Theory]. 3rd ed. Mexico City: McGraw Hill. (1995).

[38] Méndez-Sánchez M del P Peñaloza Gómez R García-Méndez M Jaenes Sánchez JC Reynoso-Sánchez LF. Percepción sobre la participación de la mujer en el deporte mexicano [Perception of women’s participation in Mexican sports]. Retos. (2023) 48:816–26. 10.47197/retos.v48.94474

[39] INMUJERES-CONADE. Mujer y Deporte. Una visión de género. Instituto Mexicano de las Mujeres y Comisión Nacional de Cultura Física y Deporte. (2006).

[40] Méndez-Sánchez MP Peñaloza R Reynoso-Sánchez LF. Equidad de género en el deporte mexicano [Gender equity in Mexican sports]. In: Pérez-Flores AM Muñoz VM Jaenes JC, editors. Valores Sociales y Deporte: Un Binomio Compacto. Madrid: Dykinson (2020). p. 62–73.

[41] Muñoz-Helú H Reynoso-Sánchez LF Peñaloza R Cruz-Morales KN Jaenes JC. Percepción del rol de la mujer en el deporte en estudiantes universitarios [Perception of women’s role in sports in university students]. In: López-Walle J Tristán J Cuevas R Ceballos A, editors. Psicología del Deporte y Ciencias Aplicadas. Monterrey: Universidad Autónoma de Nuevo León/Fondo Editorial de Nuevo León (2020). p. 255–80.

[42] World Medical Association. World medical association declaration of Helsinki: ethical principles for medical research involving human subjects. JAMA. (2013) 310:2191–4. 10.1001/jama.2013.281053 24141714

[43] Guelmami N Ben Ezzeddine L Hatem G Trabelsi O Ben Saad H Glenn JM The ethical compass: establishing ethical guidelines for research practices in sports medicine and exercise science. Int J Sport Stud Health. (2024) 7:31–46. 10.61838/kman.intjssh.7.2.4

[44] Hayes AF. Introduction to Mediation, Moderation, and Conditional Process Analysis. A Regression-based approach. 2nd ed. New York: The Guilford Press (2018).

[45] Melendro-Blanco C. Visión y propuestas con perspectiva de género para el progreso del olimpismo en Colombia [Vision and proposals with a gender perspective for the progress of olympism in Colombia]. Rev Digit Act Fís Deporte. (2022) 8:e2154. 10.31910/rdafd.v8.n1.2022.2154

[46] Longa FEA. Gender equality within the sports legal framework: a comprehensive analysis and future perspectives. Open J Soc Sci. (2025) 13:516–20. 10.4236/jss.2025.132029

[47] Dosal Ulloa R Mejía Ciro MP Capdevila Ortis L. Deporte y equidad de género. [Sport and gender equity]. Economíaunam. (2017) 14:121–33. https://www.redalyc.org/articulo.oa?id=363549671004

[48] Hang J Hijós N Moreira V. Género y alto rendimiento en Argentina: una aproximación desde las políticas públicas deportivas y las experiencias de atletas de élite [Gender and high performance in Argentina: an approach from sports public policies and the experiences of elite athletes]. In: Soto-Lagos R Moreira V, editors. Políticas Públicas del Deporte en Latinoamérica. Buenos Aires: CLACSO (2021). p. 49–66. 10.2307/j.ctv2v88d87.7

[49] Fuentes C López D’Amico R. Participación de la mujer en deportes de Alta competencia. [Women’s participation in high competition sports]. Rev Act Fís Cienc. (2018) 10(3):41–56.

[50] Santana W Oliveira M. Are the olympics up-to-date? Measures taken by the IOC to enhance gender equality in the games. Olimpianos. (2022) 6:234–50. 10.30937/2526-6314.v6.id156

[51] Guillén del Castillo M. Valores olímpicos como valores humanos. [Olympic values as human values]. Bol Real Acad Córdoba. (2016) 165:219–37.

[52] Donoso Pérez B Reina Giménez A Álvarez-Sotomayor A. Desigualdad de género en el deporte de competición: voces y reflexiones [Gender inequality in competitive sport: voices and reflections]. Retos. (2022) 47:557–64. 10.47197/retos.v47.93006

[53] Talleu C. Access for Girls and Women to Sport Practices. Strasbourg: Council of Europe. (2011). Available at: http://www.coe.int/t/DG4/EPAS/Publications/Handbook_2%20_Gender_equality_in_sport.pdf (Accessed February 08, 2024).

[54] Biernat E Krzepota J Sadowska D. Martial arts as a form of undertaking physical activity in leisure time analysis of factors determining participation of Poles. Int J Environ Res Public Health. (2018) 15:1989. 10.3390/ijerph15091989 30213133 PMC6164182

[55] Whelan G. The virtue of external goods in action sports practice. Bus Ethics Q. (2024) 35(1):84–114. 10.1017/beq.2023.37

[56] Cortez Carpio JJ. Estereotipos de género e identidad atlética en jóvenes deportistas de una universidad privada de Lima Metropolitana. [Gender stereotypes and athletic identity in young athletes from a private university of Lima Metropolitan] (bechelor thesis). Lima, Peru: Pontificia Universidad Católica del Perú. (2022).

[57] Ribeiro CHV Soares AJG Dacosta LP. Percepção sobre o legado dos megaeventos esportivos no brasil: o caso da Copa do mundo FIFA 2014 e os jogos olímpicos rio 2016. Rev Bras Ciênc Esporte. (2014) 36:447–66. 10.1590/S0101-32892014000200012

[58] Saragoça J Ferreira S Muñoz-Sánchez VM Pérez-Flores AM. Valores de ética deportiva en formación de deportistas: estudio cualitativo de un club de futbol femenino de alentejo (Portugal) [Sports ethics values in athlete training: qualitative study of a women’s volleyball club in alentejo (Portugal)]. In: Pérez-Flores AM Muñoz Sánchez VM Jaenes Sánchez JC, editors. Valores Sociales y Deporte. Madrid: Dykinson (2020). p. 39–49. 10.2307/j.ctv17hm8gm.6

[59] Tanni AA Khan MMI. Exploring the gender disparity in sports participation: a qualitative analysis of women’s limited engagement in sports in Bangladesh. Innov J Soc Sci Econ Rev. (2024) 6(1):43–51. 10.36923/ijsser.v6i1.249

[60] Jiménez Garzón LE. Interpretaciones sobre las opiniones de profesionales del deporte y afines acerca de los contenidos de la cátedra de estudios olímpicos y del deporte jean pierre de coubertin del programa de cultura física, deporte y recreación de la USTA [Interpretations of the opinions of sports and related professionals about jean pierre de coubertin chair’s contents of Olympic and sports studies from the USTA curricula physical culture, sports and recreation]. Cuerpo Cult Mov. (2012) 2:83. 10.15332/s2248-4418.2012.03-4.05

[61] Arimany Ruiz A. Enfoque y aplicaciones de la educación olímpica (Approaches and applications of Olympic education). Citius Altius Fortius. (2017) 10:1–17. 10.15366/citius2017.10.1.001

[62] Guidotti F Demarie S Ciaccioni S Capranica L. Knowledge, competencies, and skills for a sustainable sport management growth: a systematic review. Sustainability. (2023) 15:7061. 10.3390/su15097061

[63] Zhang H Powell D. Governing Olympic education: technologies of policy announcements and outsourcing. Int Rev Sociol Sport. (2023) 58:349–67. 10.1177/10126902221101993

[64] Ricardo LIC Wendt A Costa CS Mielke GI Brazo-Sayavera J Khan A Gender inequalities in physical activity among adolescents from 64 global south countries. J Sport Health Sci. (2022) 11:509–20. 10.1016/j.jshs.2022.01.007 35074485 PMC9338337

[65] Wood JT. Gendered Lives: Communication, Genderand Culture. Boston, MA: Wadsworth Cenage Learning (2011).

[66] Sánchez-Bello A. Perspectivas teóricas de género: status questionis del impacto en el sistema educativo [Theoretical perspectives on gender: status questionis of the impact on the education system]. Convergencia. (2015) 22:111–27.

[67] Etchezahar E. La construcción social del género desde la perspectiva de la teoría de la identidad social [The social construction of gender from the perspective of the social identity theory]. Cienc Docencia Tecnol. (2014) 49:128–42.

[68] Hopkins CS Hopkins C Kanny S Watson A. A systematic review of factors associated with sport participation among adolescent females. Int J Environ Res Public Health. (2022) 19:3353. 10.3390/ijerph19063353 35329041 PMC8950299

[69] Castillo C M Serra P Duclos Bastías D Castillo Retamal F. Masculinización de la matrícula universitaria en la carrera de educación física. Un análisis desde la perspectiva de género. Rev Educ. (2021) 46(1):299–316. 10.15517/revedu.v46i1.47576

[70] UNESCO. From Access to Empowerment: UNESCO’s Strategy for Gender Equality in and through Education 2019-2025 (2019).

