# Supplementary material for: In the perception of the Olympic movement and gender equity in sport, are gender and sport practice determining factors?
Source: Front Sports Act Living. 2025 Mar 31;7:1564617. doi: 10.3389/fspor.2025.1564617 (PMC11994579; doi:10.3389/fspor.2025.1564617)
Supplement: Supplementary file 1 [file Datasheet1.pdf]

Supplementary Material

1 Original (Spanish) and translated items of the Olympism vision and its educational repercussions questionnaire

Original reference: Gomez-Marmol, A., Sanchez-Alcaraz, B.J., Molina, J.M. & Bazaco, M.J. (2016). Estudio preliminar para el diseno y validacion del "Cuestionario sobre la vision del Olimpismo y sus repercusiones educativas (CUVOREDU)". ***Rexe: Revista de Estudios y Experiencias en Educacion*****, 15(28), 129-144.**

Escala sobre conocimientos y percepción del Movimiento Olímpico

Bloque I. Conocimiento sobre Olimpismo

LEA ATENTAMENTE CADA CUESTIÓN ANTES DE RESPONDER

Sólo existe una opción correcta

Marque con una X la respuesta que considere verdadera

1. Ciudad primeros JJOO (Atenas)
   a) Roma b) París c) Atenas d) No lo sé
2. Año primeros JJOO (1986)
   a) 1842 b) 1896 c) 1908 d) No lo sé
3. Ciudad JJOO 2016 (Río de Janeiro)
   a) Londres b) Río de Janeiro c) Vancouver d) No lo sé
4. Ciudad JJOO 2008 (Pekin)
   a) Sydney b) Turín c) Pekín d) No lo sé
5. Autor "lo importante no es ganar sino participar" (Monseñor Talbot)
   a) Pierre de Coubertin b) Monseñor Talbot c) Padre Didón d) Profesor Sloane
6. ¿Qué es la carta Olímpica? (La norma fundamental del Olimpismo)
   a) La misiva que lee un deportista en la ceremonia de inauguración de los JJOO
   b) La Norma fundamental del Olimpismo
   c) Las Memorias del fundador de los JJOO Modernos
   d) El texto protocolario que clausura los JJOO
7. Restaurador de los JJOO modernos (Pierre de Coubertin)
   a) Francisco Amorós b) Pierre de Coubertin c) Vittorio da Feltre d) Werner Jaeger
8. Significado de los JJOO modernos (Unión de los 5 continentes)
   a) La unión de los cinco continentes y el encuentro de los atletas del mundo en los Juegos Olímpicos
   b) Las cinco religiones más universales
   c) Las ciudades-estados griegas, que competían en los JJOO antiguos

d) La práctica universal del deporte en todos los países del mundo

9. Actual presidente del COI (Jaques Rogge)
   a) Jaques Rogge b) Joseph Blatter c) Juan Antonio Samaranch d) Alberto de Mónaco
10. ¿Cuál es el lema olímpico? (Citius, Altius, Fortius)
   a) Mens sana in corpore sano
   b) Citius, Altius, Fortius
   c) Mens fervida in corpore lacertoso
   d) Lo importante no es ganar sino participar

Bloque II. Opinión sobre los posibles riesgos y/o amenazas a los que se enfrenta el Movimiento Olímpico

A continuación encontrará una serie de cuestiones que hace referencia al Movimiento Olímpico

LEA ATENTAMENTE CADA CUESTIÓN ANTES DE RESPONDER.

Por favor, marque sólo una respuesta para cada pregunta. Marque con un círculo su respuesta

Es necesario que responda a todos los apartados.

No existen respuestas verdaderas o falsas, buenas o malas, es solo su opinión.

El resultado de este trabajo está vinculado con la sinceridad de su opinión.

Junto a cada afirmación encontrará 5 (cinco) opciones, en las que cada número representa una opinión diferente:

0- No estoy NADA de acuerdo
1- Estoy POCO de acuerdo
2- Estoy MEDIANAMENTE de acuerdo
3- Estoy BASTANTE de acuerdo
4- Estoy MUCHO de acuerdo

2.1. Amenazas o riesgos del movimiento olímpico

En mi opinión el/la…

11. Comercialización como factor negativo del Movimiento Olímpico en el futuro
12. Dopaje como factor negativo del Movimiento Olímpico en el futuro
13. Falta de fair-play como factor negativo del Movimiento Olímpico en el futuro
14. Nacionalismo como factor negativo del Movimiento Olímpico en el futuro
15. Gigantismo como factor negativo del Movimiento Olímpico en el futuro
16. Terrorismo como factor negativo del Movimiento Olímpico en el futuro
17. Profesionalización deportiva como factor negativo del Movimiento Olímpico en el futuro
18. Boicots como factor negativo del Movimiento Olímpico en el futuro
19. Amenaza de la corrupción como factor negativo del Movimiento Olímpico en el futuro

2.2. Percepción de los valores que transmite el Movimiento Olímpico

Escala de respuesta

0- No estoy NADA de acuerdo
1- Estoy POCO de acuerdo
2- Estoy MEDIANAMENTE de acuerdo
3- Estoy BASTANTE de acuerdo
4- Estoy MUCHO de acuerdo

20. ¿En qué medida los JJOO son un buen ejemplo para la juventud?
21. ¿En qué medida lo más importante en los JJOO es ganar? (se invierte)
22. ¿En qué medida valora que los Juegos Olímpicos son el evento deportivo más ipmortante a nivel mundial?
23. ¿En qué medida para ganar una medalla olímpica hay que doparse? (Se invierte)
24. ¿En qué medida es interesante organizar un "día olímpico" para el fair-play en escolares?
25. ¿En qué medida los JJOO transmiten fair-play entre los espectadores?

2.3. Significado del Olimpismo

Seguidamente le propondremos 10 (diez) opciones de lo que puede significar para usted el Olimpismo.

LEA ATENTAMENTE TODAS LAS OPCIONES ANTES DE RESPONDER

Por favor, marque sólo una respuesta para cada pregunta. Marque con un círculo su respuesta

Junto a cada afirmación encontrará 5 (cinco) opciones, en las que cada número representa una opinión diferente:

0- No estoy NADA de acuerdo
1- Estoy POCO de acuerdo
2- Estoy MEDIANAMENTE de acuerdo
3- Estoy BASTANTE de acuerdo
4- Estoy MUCHO de acuerdo

En su opinión, el Olimpismo es:

26. El Olimpismo es una filosofía
27. El Olimpismo es una ideología
28. El Olimpismo es un estado del espíritu
29. El Olimpismo es un ideal humanista
30. El Olimpismo es una cuestión de dinero (Se invierte)
31. El Olimpismo es una hipocresía (Se invierte)
32. El Olimpismo es una utopía (Se invierte)

33. El Olimpismo es un mito (Se invierte)
34. El Olimpismo es una actitud moral
35. El Olimpismo es un sistema corrupto (Se invierte)

Scale on Knowledge and Perception of the Olympic Movement

Block I. Knowledge about Olympism

PLEASE READ EACH QUESTION CAREFULLY BEFORE ANSWERING

There is only one correct answer.

Mark the option you consider correct with an X.

1. City of the first Olympic Games (Athens)
   a) Rome b) Paris c) Athens d) I don't know

2. Year of the first Olympic Games (1896)
   a) 1842 b) 1896 c) 1908 d) I don't know

3. Host city of the 2016 Olympic Games (Rio de Janeiro)
   a) London b) Rio de Janeiro c) Vancouver d) I don't know

4. Host city of the 2008 Olympic Games (Beijing)
   a) Sydney b) Turin c) Beijing d) I don't know

5. Author of the phrase "The important thing is not to win but to take part" (Monsignor Talbot)
   a) Pierre de Coubertin b) Monsignor Talbot c) Father Didon d) Professor Sloane

6. What is the Olympic Charter? (The fundamental rule of Olympism)
   a) The letter read by an athlete at the opening ceremony of the Olympic Games.
   b) The fundamental rule of Olympism.
   c) The memoirs of the founder of the Modern Olympic Games.
   d) The official text that closes the Olympic Games.

7. Restorer of the Modern Olympic Games (Pierre de Coubertin)
   a) Francisco Amorós
   b) Pierre de Coubertin
   c) Vittorio da Feltre
   d) Werner Jaeger

8. Meaning of the Modern Olympic Games (Union of the 5 continents)
   a) The union of the five continents and the gathering of athletes from around the world at the Olympic Games.

b) The five most universal religions.
c) The Greek city-states that competed in the ancient Olympic Games.
d) The universal practice of sports in all countries worldwide.

9. Current IOC President (Jacques Rogge)
a) Jacques Rogge b) Joseph Blatter c) Juan Antonio Samaranch d) Albert of Monaco

10. What is the Olympic motto? (Citius, Altius, Fortius)
a) Mens sana in corpore sano
b) Citius, Altius, Fortius
c) Mens fervida in corpore lacertoso
d) The important thing is not to win but to participate

2.1. Opinion about Possible Risks and/or Threats to the Olympic Movement

The following questions refer to the Olympic Movement.
PLEASE READ EACH QUESTION CAREFULLY BEFORE ANSWERING.

- Please mark only one response per question by circling your answer.
- It is essential that you answer all questions.
- There are no right or wrong answers; it is only your opinion.
- The results of this survey depend on the sincerity of your answers.

Next to each statement, you will find five (5) options, where each number represents a different opinion:
0 - I STRONGLY DISAGREE
1 - I SLIGHTLY DISAGREE
2 - I MODERATELY AGREE
3 - I FAIRLY AGREE
4 - I STRONGLY AGREE

2.1. Threats or Risks to the Olympic Movement

In my opinion, the following are negative factors for the future of the Olympic Movement:
11. Commercialization as a negative factor for the future of the Olympic Movement.
12. Doping as a negative factor for the future of the Olympic Movement.
13. Lack of fair play as a negative factor for the future of the Olympic Movement.
14. Nationalism as a negative factor for the future of the Olympic Movement.
15. Gigantism (excessive growth of the Games) as a negative factor for the future of the Olympic Movement.
16. Terrorism as a negative factor for the future of the Olympic Movement.
17. Professionalization of sports as a negative factor for the future of the Olympic Movement.
18. Boycotts as a negative factor for the future of the Olympic Movement.
19. Corruption as a negative factor for the future of the Olympic Movement.

2.2. Perception of the Values Promoted by the Olympic Movement

Answer Scale:
0 - I STRONGLY DISAGREE
1 - I SLIGHTLY DISAGREE
2 - I MODERATELY AGREE
3 - I FAIRLY AGREE
4 - I STRONGLY AGREE

20. To what extent do you think the Olympic Games are a good example for youth?

21. To what extent do you think winning is the most important aspect of the Olympic Games? *(Reversed item)*

22. To what extent do you value the Olympic Games as the most important sporting event in the world?

23. To what extent do you think it is necessary to dope to win an Olympic medal? *(Reversed item)*

24. To what extent do you think organizing an “Olympic Day” to promote fair play in schools is interesting?

25. To what extent do you think the Olympic Games promote fair play among spectators?

2.3. Meaning of Olympism

Next, you will find ten (10) statements that refer to the meaning of Olympism.
PLEASE READ ALL OPTIONS CAREFULLY BEFORE ANSWERING.

- Please mark only one response per statement by circling your answer.

- Next to each statement, you will find five (5) options, where each number represents a different opinion:
  0 - I STRONGLY DISAGREE
  1 - I SLIGHTLY DISAGREE
  2 - I MODERATELY AGREE
  3 - I FAIRLY AGREE
  4 - I STRONGLY AGREE

In your opinion, Olympism is:
26. Olympism is a philosophy.
27. Olympism is an ideology.
28. Olympism is a state of mind.
29. Olympism is a humanistic ideal.
30. Olympism is a matter of money. *(Reversed item)*
31. Olympism is hypocrisy. *(Reversed item)*
32. Olympism is a utopia. *(Reversed item)*
33. Olympism is a myth. *(Reversed item)*

34. Olympism is a moral attitude.
35. Olympism is a corrupt system. *(Reversed item)*

2 Original (Spanish) and translated versión of the Attitudes towards Women's Participation in Sport Scale

Original reference: Méndez Sánchez, M. del P., Peñaloza Gómez, R., García Méndez, M., Jaenes Sánchez, J. C., & Reynoso Sánchez, L. F. (2023). Percepción Sobre la Participación de la Mujer en el Deporte Mexicano (Perception about Participation of Women in Mexican Sports). ***Retos*****, 48, 816–826. https://doi.org/10.47197/retos.v48.94474**

Escala de Actitudes hacia la Participación de la Mujer en el Deporte

A continuación, se presentan una serie de afirmaciones que tiene como objetivo identificar tu percepción por lo que te pedimos respondas con total sinceridad. No hay respuestas correctas o incorrectas, elije solamente la respuesta que mejor refleje tu opinión. Asegúrate de no dejar ninguna sin responder. Responde de acuerdo con la siguiente escala:

Totalmente en Desacuerdo (1); en Desacuerdo (2); Indeciso (3); de Acuerdo (4); Totalmente de Acuerdo (5).

Items:

1. Las mujeres pueden desarrollar sus capacidades físicas tanto como los hombres
2. Las mujeres pueden tener una carrera deportiva estable.
3. Las mujeres tienen la misma dedicación al deporte que los hombres.
4. Una mujer puede llegar tan lejos como un hombre si así se lo propone.
5. Mi familia opina que una mujer puede ser tan buena deportista como un hombre.
6. La mujer tiene el mismo derecho que el hombre a dedicarse al deporte.
7. En los deportes a la mujer se le trata con respeto.
8. El trato a la mujer en los deportes es igual al de los hombres.
9. Las escuelas apoyan de la misma forma a los deportistas de ambos sexos.
10. Las mujeres y los hombres deportistas reciben el mismo apoyo de las instituciones deportivas.
11. La mujer y el hombre reciben el mismo apoyo de sus familias para dedicarse al deporte.
12. La mujer deportista recibe los incentivos económicos que merece.
13. Se ofrece un número equitativo de becas para el deporte a hombres que a mujeres.
14. En México existen las instalaciones adecuadas para las mujeres deportistas.

Factores e interpretación

Factor 1: Percepción social de la mujer en el deporte (Items: 1 -6). Interpretación: Puntajes más altos evidencian una percepción positiva de la participación de la mujer en el deporte.

Factor 2: Percepción de equidad en el deporte (7.11). Interpretación: Puntajes más altos evidencian una percepción de mayor equidad de género en el deporte.

Factor 3: Apoyo social para la mujer en el deporte (12-14). Interpretación: Puntajes más altos evidencian una percepción menor o bajo apoyo para la mujer en el deporte.

Scale of Attitudes Toward Women's Participation in Sports

Below are a series of statements designed to identify your perception of women's participation in sports. Please answer as honestly as possible. There are no right or wrong answers — simply select the response that best reflects your opinion. Make sure not to leave any questions unanswered. Respond according to the following scale:
Strongly Disagree (1); Disagree (2); Neutral (3); Agree (4); Strongly Agree (5).

Items:

1. Women can develop their physical abilities as much as men.
2. Women can have a stable sports career.
3. Women have the same dedication to sports as men.
4. A woman can go as far as a man if she sets her mind to it.
5. My family believes that a woman can be as good an athlete as a man.
6. Women have the same right as men to pursue a career in sports.
7. Women are treated with respect in sports.
8. Women receive the same treatment in sports as men.
9. Schools provide equal support to male and female athletes.
10. Female and male athletes receive the same support from sports institutions.
11. Women and men receive the same support from their families to pursue a sports career.
12. Female athletes receive the financial incentives they deserve.
13. An equitable number of sports scholarships are offered to both men and women.
14. In Mexico, there are adequate sports facilities for female athletes.

Factors and Interpretation

Factor 1: Social Perception of Women in Sports (Items 1-6). Interpretation: Higher scores indicate a positive perception of women's participation in sports.

Factor 2: Perception of Gender Equity in Sports (Items 7-11). Interpretation: Higher scores indicate a perception of greater gender equity in sports.

Factor 3: Social Support for Women in Sports (Items 12-14). Interpretation: Higher scores indicate a perception of lower or insufficient support for women in sports.
